# Draft Genome Sequences of Neofusicoccum parvum Strains Isolated from Mango and Rice-Paper Plant (Tetrapanax papyrifer)

**DOI:** 10.1128/mra.00259-23

**Published:** 2023-05-23

**Authors:** Akira Ashida, Maurizio Camagna, Ikuo Sato, Sotaro Chiba, Aiko Tanaka, Daigo Takemoto

**Affiliations:** a Graduate School of Bioagricultural Sciences, Nagoya University, Nagoya, Japan; University of California, Riverside

## Abstract

Neofusicoccum parvum is a polyxenous phytopathogenic fungus that infects important fruits, such as grapes and mangoes. Here, we report the genome sequences of N. parvum strains that were isolated from mango in Okinawa, Japan (strain PPO83), and an invasive weed (rice-paper plant [Tetrapanax papyrifer]) in Nagoya, Japan (strain NSSI1).

## ANNOUNCEMENT

Neofusicoccum parvum is a polyxenous phytopathogenic fungus that causes severe diseases in important fruit crops, such as grapes and mangoes. N. parvum infects tree trunks and fruit stems through wounds after pruning, causing wilt and necrosis of trees or fruits (1–3). Pruning is essential in orchards with intensive cultivation of high-unit-value fruit trees, to control vigor and fruit quality, but this process contributes to increasing the risk of N. parvum infection. Infection of N. parvum through fruit stalks is a major problem of mango production, causing internal rotting of the fruits by the time they reach consumers (3).

We previously isolated N. parvum strain NSSI1 from leaf blight symptoms on a rice-paper plant (Tetrapanax papyrifer) in Nagoya, Japan. Rice-paper plant is a woody plant that is native to Taiwan and has become naturalized in temperate and tropical zones around the world. Both strain NSSI1 and another strain, namely, strain PPO83, which was isolated from mango stem in Okinawa, Japan, are pathogenic to grape, mango, and several weeds around arable land (4). The N. parvum strains were deposited at the Research Center of Genetic Resources, National Agriculture and Food Research Organization (Tsukuba, Japan), with accession numbers MAFF247736 (NSSI1) and MAFF247737 (PPO83). Because some polyxenous pathogens have the abilities to metabolize a variety of antimicrobial compounds (phytoalexins) produced by host plants (5–7), the genomes of N. parvum strains are expected to contain detoxification genes that are essential for their infection of plants. Here, we report the draft genome sequences of strains NSSI1 and PPO83. Both strains were grown on potato dextrose agar (PDA) for 3 days and then in liquid potato dextrose broth (PDB) for 24 h at 28°C on a rotary shaker at 150 rpm for genomic DNA extraction from their mycelia using the DNeasy plant minikit (Qiagen, Germany). Whole-genome sequencing libraries were prepared with the DNA preparation (M) tagmentation kit (Illumina, San Diego, CA, USA) and sequenced on a NovaSeq 6000 instrument (Illumina) using the NovaSeq 6000 SP reagent kit v1.5 with 2 × 150-bp reads. After quality filtering and trimming with fastp v0.20.0 with options to remove adaptors and low-quality reads (8), paired-end data sets were assembled *de novo* using SPAdes v3.15.3 with the –careful option (9). Assembly quality was assessed with QUAST v4.4 (10). The summary of data for the genome assemblies is presented in Table 1. Prediction of the protein-encoding genes was performed with BRAKER2 (11), using the annotated protein sequences of N. parvum UCR-NP2 (12) as the input for model hints. The qualities of the assembled genomes were estimated using benchmarking universal single-copy orthologs (BUSCO) with the hypocreales_odb10 and dothideomycetes_odb10 data sets (13). Using the Hypocreales data set, the protein sets of NSSI1 and PPO83 were found to have BUSCO scores of 90.2%, with a total of 230 complete BUSCOs for both strains (Table 1).

**TABLE 1:** Summary of Neofusicoccum parvum genome assemblies and annotations

Parameter	Finding for strain:	
	NSSI1	PPO83
Host plant	Tetrapanax papyrifer	Mangifera indica
No. of filtered read pairs	19,934,690	37,049,625
*N*_50_ (bp)	535,253	233,081
GC content (%)	56.9	56.7
Total size (bp)	43,149,159	43,240,122
No. of contigs	377	566
Sequencing coverage (×)	133	252
No. of genes	13,549	13,394
Results from BUSCO analysis		
With hypocreales_odb10 data set		
Proportion of complete BUSCOs (%)	90.2	90.2
No. of BUSCOs	230	230
No. of missing BUSCOs	8	7
No. of fragmented BUSCOs	17	18
With dothideomycetes_odb10 data set		
Proportion of complete BUSCOs (%)	86.8	86.5
No. of BUSCOs	3,284	3,276
No. of missing BUSCOs	164	171
No. of fragmented BUSCOs	338	339

### Data availability.

The genome sequences of the N. parvum strains were deposited in DDBJ/EMBL/GenBank under accession numbers BSXH01000001 to BSXH01000377 (NSSI1) and BSXG01000001 to BSXG01000566 (PPO83). The raw sequencing reads were submitted to the DDBJ Sequence Read Archive (DRA) under accession numbers DRR452881 (NSSI1) and DRR452882 (PPO83).
